# Palmitic Acid Induced a Long-Lasting Lipotoxic Insult in Human Retinal Pigment Epithelial Cells, which Is Partially Counteracted by TRAIL

**DOI:** 10.3390/antiox11122340

**Published:** 2022-11-26

**Authors:** Domenico Sergi, Enrico Zauli, Fabio Casciano, Paola Secchiero, Giorgio Zauli, Matteo Fields, Elisabetta Melloni

**Affiliations:** 1 Department of Translational Medicine, University of Ferrara, 44121 Ferrara, Italy; 2 Department of Translational Medicine and LTTA Centre, University of Ferrara, 44121 Ferrara, Italy; 3 Interdepartmental Research Center for the Study of Multiple Sclerosis and Inflammatory and Degenerative Diseases of the Nervous System, University of Ferrara, 44121 Ferrara, Italy; 4 King Khaled Eye Specialistic Hospital, Riyadh 11462, Saudi Arabia

**Keywords:** ARPE-19, TRAIL, lipotoxicity, palmitic acid, type 2 diabetes mellitus, retinal pigment epithelial cells

## Abstract

Hyperglycaemia and increased circulating saturated fatty acids are key metabolic features of type 2 diabetes mellitus (T2DM) that contribute to diabetic retinopathy pathogenesis. Contrarily, tumor necrosis factor (TNF)-related apoptosis-inducing ligand (TRAIL) has been shown to improve or prevent T2DM. This study aimed at investigating the effect of TRAIL in an in vitro model of human retinal pigment epithelium: the ARPE-19 cell line, treated with palmitic acid (PA) in the presence of high glucose concentration. PA caused a drop in cellular metabolic activity and cell viability as well as an increase in apoptosis rates, which were paralleled by an upregulation of reactive oxygen species (ROS) generation as well as mitochondrial fragmentation. Despite ARPE-19 cells expressing TRAIL-R2 at the cell surface, TRAIL failed to counteract the cytotoxic effects of PA. However, when TRAIL was used alongside PA and then removed or used alone following PA challenge, it partially attenuated PA-induced lipotoxicity. This effect of TRAIL appeared to rely upon the modulation of inflammation and ROS production. Thus, TRAIL exerted a trophic effect on ARPE-19 cells, which became evident only when the lipotoxic insult was removed. Nevertheless, whether recombinant TRAIL might have a therapeutic potential for the treatment of diabetic retinopathy requires further investigation.

## 1. Introduction

About 60% of patients affected by type 2 diabetes mellitus (T2DM) develop diabetic retinopathy (DR) in their lifetime [1], with diabetic dyslipidemia contributing to DR progression. Diabetic mice have over three-times the retinal fatty acid (FA) content of healthy controls and palmitic acid (PA) is elevated above other free FAs in the circulation and tissues of diabetic patients and animal models of diabetes [2,3,4]. Considering the cytotoxic effects exerted by PA in a variety of cell models [5,6,7], its increase in the circulation may contribute to DR in concert with diabetic hyperglycemia.

Retinal pigment epithelium (RPE) is the specialized epithelium lying in the outermost layer of the neural retina. Recent studies have shown that, similarly to other neuronal cell types [3,8,9,10,11], RPE cells might also be involved both in the early phase as well as in the more advanced phase of DR development and progression [12]. In this regard, the increased circulating PA levels observed in the context of T2DM may contribute to DR by eliciting a cytotoxic effect on RPE cells. Indeed, PA has been found to induce injury in vitro in retinal ganglion cells [8] as well as in Müller cells [10] and in retinal pigment epithelial cells [12,13].

Intraocular injections of anti-vascular endothelial growth factor (VEGF) agents are currently the most effective therapy for inhibiting the angiogenesis seen in DR, but less than 50% of patients have improved vision after 1–2 years of anti-VEGF injections [14,15]. In general, the current therapies for DR mainly target the ocular neovascularization at the later stages of DR, when visual function is already damaged and difficult to restore. Therefore, it is important to investigate the pathological process in the neural retina at the early stages of DR in order to develop new therapeutic strategies.

In this context, tumor necrosis factor (TNF)-related apoptosis-inducing ligand (TRAIL) may represent a potential therapeutic candidate to tackle DR. It belongs to the TNF superfamily of proteins and regulates multiple fundamental cellular processes, ranging from apoptosis of transformed cell lines to cell proliferation. A growing body of experimental and clinical evidence suggests that TRAIL plays an important role in the pathophysiology of T2DM [16,17]. In particular, it has been shown that insulin resistance, the hallmark of T2DM, and its associated cardio-metabolic aberrations can be accelerated and exacerbated by TRAIL deletion [18,19]. On the contrary, diabetes was reported to be effectively prevented and ameliorated by recombinant TRAIL treatment [20]. Additionally, in a clinical setting, it has been shown that circulating soluble TRAIL levels are significantly reduced in patients in both T1DM and T2DM as well as in diabetes-related macro- and microvascular complications [21,22,23,24]. Conversely, it has also been shown that the serum TRAIL levels of patients with T2DM progressively increase upon antidiabetic treatment [25]. However, the effects of TRAIL are not limited to the prevention and amelioration of T2DM per se, as they may also apply to DR. Indeed, TRAIL deficiency has been associated with a delayed regression of retinal neovascularization [26] and a drop in soluble TRAIL has been reported in the conjunctival sac fluid [27] and in vitreous samples [28] of patients with DR. Despite the putative beneficial effects of TRAIL, it remains to be investigated whether it is able to counteract the lipotoxic effects of a saturated fatty acids overload on RPE cells and its mechanisms of action.

Thus, the aim of this study was to evaluate the effect of TRAIL on the lipotoxic damage induced by PA on RPE, using the ARPE-19 cell line as a model system.

## 2. Materials and Methods

### 2.1. Cell Culture

ARPE-19, a retinal pigment epithelial (RPE) cell line derived from the normal eyes of a deceased 19-year-old male, was obtained from American Type Culture Collection (ATCC, Manassas, VA, USA). Cells were maintained at 37 °C under a 5% CO_2_ and 90% relative humidity atmosphere in Dulbecco’s Modified Eagle Medium: Nutrient Mixture F-12 (DMEM/F-12) containing 10% fetal bovine serum (FBS), 2 mM L-glutamine, 100 U/mL penicillin and 100 mg/mL streptomycin (all from Gibco, Grand Island, NY, USA).

### 2.2. Cell Treatments

**Figure 1:**
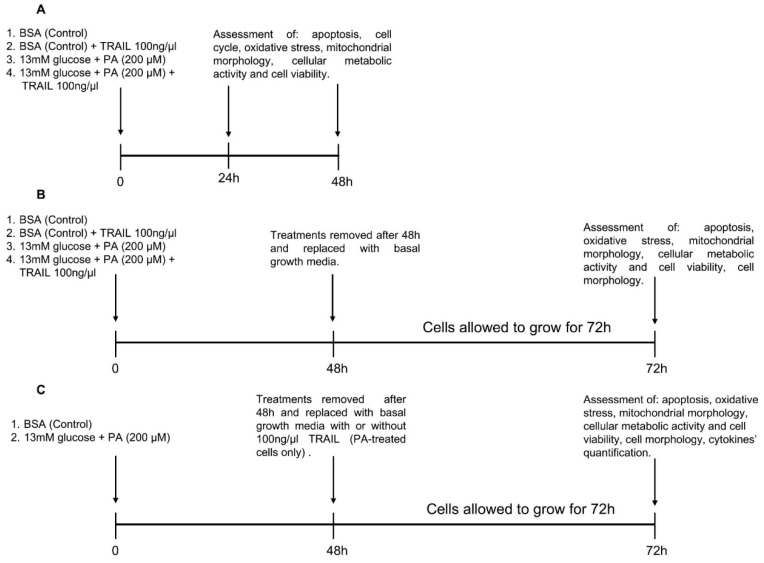
Schematic representation of recovery experiments. ARPE-19 cells were treated with bovine serum albumin (BSA) in the presence or absence of tumor necrosis factor (TNF)-related apoptosis-inducing ligand (TRAIL) as well as palmitic acid (PA) and high glucose concentration (13 mM glucose) with or without TRAIL. In the first sets of experiments (**A**) after 24 or 48 h of treatment the indicated assays were assessed. In the “recovery experiments” (**B**,**C**), treatments were removed after 48 h and cells were incubated with growth media (**B**) or with growth media in the absence or presence of TRAIL (PA-treated cells only) (**C**) for additional 72 h and then assessed as detailed in the Materials and Methods section.

Cells were grown in either 6-, 24- or 96-well plates, depending on the performing assay, and treated with 0.5 µM fatty-acid-free bovine serum albumin (BSA, Sigma Aldrich, St. Louis, MO, USA), used as vehicle, in low-glucose (5.5 mM) or high-glucose (13 mM) Dulbecco’s Modified Eagle Medium (DMEM, Gibco) supplemented with 1% FBS (Gibco) in the presence or absence of PA (PA, Sigma Aldrich) and/or 100 ng/mL recombinant TRAIL, prepared as previously described [29], for 24 or 48 h, following the experimental design schematized in Figure 1A.

To define the most suitable concentration of PA to induce lipotoxicity, ARPE-19 cells were preliminarily treated with PA at 50, 100 and 200µM, in high- or low-glucose DMEM for comparative evaluations and 200µM was selected for the experimental procedures described hereafter.

For the cell treatment, PA was conjugated to low-endotoxin, fatty-acid-free BSA as described previously [30]. Briefly, PA was dissolved in 0.1 M NaOH in a water bath at 70 °C to yield a final concentration of 20 mM. In parallel, BSA was dissolved in serum and penicillin–streptomycin-free DMEM at a final concentration of 0.5 mM. After its solubilization, PA was mixed with BSA to obtain a 1:4 molar ratio (PA 2 mM: BSA 0.5 mM). The solution was then vortexed and incubated at 55 °C for PA–BSA conjugation to occur. Finally, the PA–BSA mix was cooled to room temperature, filter sterilized and stored at 20 °C until use.

In a different subset of experiments referred to as “recovery experiments”, ARPE-19 cells were initially exposed to the same treatments described above for 48 h and then maintained in treatment-free DMEM/F-12 containing FBS and penicillin–streptomycin for additional 72 h (Figure 1 B). Additionally, in a subgroup of PA-treated cells, after treatment removal, cells were exposed to growth media in the presence of TRAIL (100 ng/mL) (Figure 1C).

### 2.3. Cell Viability, Proliferation and Apoptosis Evaluation Assays

To assess the impact of nutrient overload on cell viability and to evaluate whether TRAIL was able to increase cell proliferative capacity in the recovery experiments, cell viability/proliferation was evaluated on ARPE-19 by Trypan blue dye exclusion and MTT colorimetric assay (Roche Diagnostics Corporation, Indianapolis, IN, USA) following the manufacturer’s instructions. MTT assay quantification was performed reading the absorbance at 570 nm using a TECAN Infinite^®^ M Plex microplate reader (Tecan Trading AG, Männedorf, Switzerland, CH).

The MTT assay was complemented by the assessment of apoptosis. In this regard, the percentage of apoptotic cells was determined by a BD FACSCalibur flow cytometer (BD Biosciences, San Josè, CA, USA) after double staining with an Annexin V-FITC/propidium iodide kit (Beckman Coulter Inc., Brea, CA, USA), following the manufacturer’s instructions and as previously detailed [31]. Apoptosis data analysis was performed using the FlowJo software, version 9.9.6 (Tree Star, Ashland, OR, USA).

### 2.4. Evaluation of Real-Time Effect of Lipotoxicity and TRAIL Treatments

The time-course effects of PA and of TRAIL treatments on ARPE-19 cell line were evaluated using an xCELLigence RTCA DP Instrument (F. Hoffmann-La Roche SA, Basel, Switzerland) that registers impedance values related to cell viability and proliferation in real time (in these experiments every 15 min), converting them in the “Cell Index” (CI) adimensional parameter.

For this purpose, 5 × 10^3^ cells were seeded onto 16-well E-plates (Agilent, Santa Clara, CA, USA) in 200 µL of DMEM/F12 complete medium and cultured in presence of 5% CO_2_ and 90% relative humidity at 37 °C. Following the manufacturer’s suggestions, after approximately 24 h from the seeding, the cells were treated with 0.5 µM fatty-acid-free BSA or 200 μM PA together with or without 100 ng/mL recombinant TRAIL. After 48 h of treatment, the medium was replaced with fresh complete DMEM/F12 and, where indicated, treated with 100 ng/mL TRAIL.

### 2.5. Assessment of Mitochondrial Morphology

ARPE-19 cells were grown on glass coverslips placed in 24-well plates and seeded at a density of 6 × 10^4^/well. After treatments with BSA, PA or TRAIL for the indicated times, medium was removed and cells were washed with phosphate-buffered saline (PBS). Cells were then incubated for 20 min in FBS and penicillin–streptomycin-free medium containing 100 nM MitoTracker^TM^ Red CMXRos (Thermo Fisher Scientific, Waltham, MA, USA), a fluorescent dye which accumulates in the mitochondria in a membrane-potential-dependent fashion. After the staining, cells were washed with PBS and fixed using 4% paraformaldehyde (Sigma-Aldrich) for 15 min at room temperature. Finally, the coverslips were mounted on slides using glycerol containing 1,4-diazabicyclo [2.2.2] octane (DABCO) to retard fading (both from Sigma-Aldrich). Cell images were acquired by a Nikon Upright Microscope Eclipse Ci-S equipped with a DS-Qi2Mc digital camera using the NIS-ELEMENTS D software Version 5.11.00 (all from Nikon, Tokyo, Japan).

### 2.6. Analysis of Oxidative Stress

Intracellular reactive oxygen species were assayed using CellROX^TM^ Green Reagent (Thermo Fisher Scientific) according to the manufacturer protocol. Briefly, cells growing in 6-well plates were treated as described above or exposed to 10 µM menadione for 30 min as a positive control. Successively, cells were incubated for 30 min with 10µM CellROX^TM^ Green Reagent, washed with PBS and trypsinized. To remove dead cells from the analysis, cells were further incubated with LIVE/DEAD™ Fixable Far Red Dead Cell Stain Kit (Thermo Fisher Scientific) at 4 °C for 10 min. Samples were acquired using BD FACSCalibur Flow Cytometer (BD Biosciences, San Josè, CA, USA) and analyzed with the FlowJo software, version 9.9.6 (Tree Star, Ashland, OR, USA).

### 2.7. Evaluation of Surface Expression of TRAIL Receptors

The ARPE-19 surface expression of TRAIL and TRAIL receptors was evaluated using a BD FACSCalibur flow cytometer (BD Biosciences). For this purpose, cells were stained as previously described [32]. Briefly, cells treated for 48 h with 0.5 µM BSA (Sigma Aldric) and PA in high-glucose DMEM (Gibco) supplemented with 1% FBS (Gibco), as described before, were harvested and stained with PE-conjugated MoAbs anti-human TRAIL-R1, TRAIL-R2, TRAIL-R3 and TRAILR4 (all from R&D Systems, Minneapolis, MN, USA) and LIVE/DEAD™ Fixable Far Red Dead Cell Stain Kit (Thermo Fisher Scientific).

### 2.8. Cytokines’ Quantification

The cytokines’ analysis was performed in duplicate at the indicated time point on cell supernatants, frozen and thawed only once, using the 27-Bio-Plex assay (BioRad Laboratories, Milan, Italy) and read on MAGPIX instrument (Merck Millipore) equipped with the MILLIPLEX-Analyst Software (Merck Millipore) using a five-parameter nonlinear regression formula to compute sample concentrations from the standard curves. Quality controls provided in the multiplex kits were used to validate the assay performance.

The cytokines analyzed were: interleukin (IL)-1ra, IL-1β, IL-2, IL-4, IL-5, IL-6, IL-7, IL-8, IL-9, IL-10, IL-12 (p70), IL-13, IL-15, IL-17A, eotaxin, Granulocyte Colony-Stimulating Factor (G-CSF), Granulocyte–Macrophage Colony-Stimulating Factor (GM-CSF), interferon (IFN)-γ, Monocyte Chemotactic Protein-1 (MCP-1), Macrophage Inflammatory Protein (MIP)-1α, MIP-1β, RANTES (Regulated And Normal T cells Expressed and presumably Secreted), Tumor Necrosis Factor-α (TNF-α), VEGF (Vascular-Endothelial Growth Factor), PDGF (Platelet-Derived Growth Factor)-BB, IP-10 (Interferon gamma-induced Protein 10) and FGF (Fibroblast Growth Factor).

### 2.9. Statistical Analysis

Data are expressed as mean ± SEM of at least three independent experiments. Differences between two conditions were analyzed by Student’s t-test whereas comparisons between three or more treatments were performed using one-way ANOVA followed by Tukey’s post hoc test to correct for multiple comparisons. A *p*-value <0.05 was considered statistically significant. Statistical analyses were performed using GraphPad Prismfor Windows (version 8, GraphPad Software, San Diego, CA, USA).

## 3. Results

### 3.1. Effects of High Glucose Concentration and PA on ARPE-19 Cell Viability

**Figure 2:**
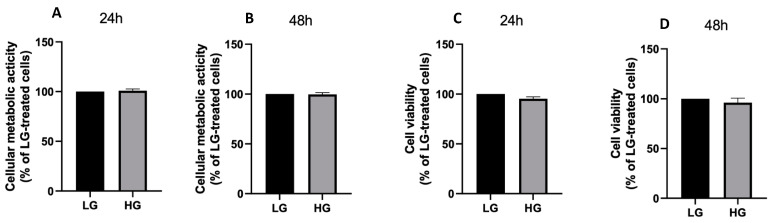
Cellular metabolic activity and viability in response to high glucose concentration. ARPE-19 cell metabolic activity (**A**,**B**) and cell viability (**C**,**D**) assessed after 24 (**A**,**C**) and 48 h (**B**,**D**) incubation with low glucose (LG, 5 mM glucose) or high glucose concentration (HG, 13 mM glucose). Data are expressed as mean ± SEM of at least three independent experiments.

**Figure 3:**
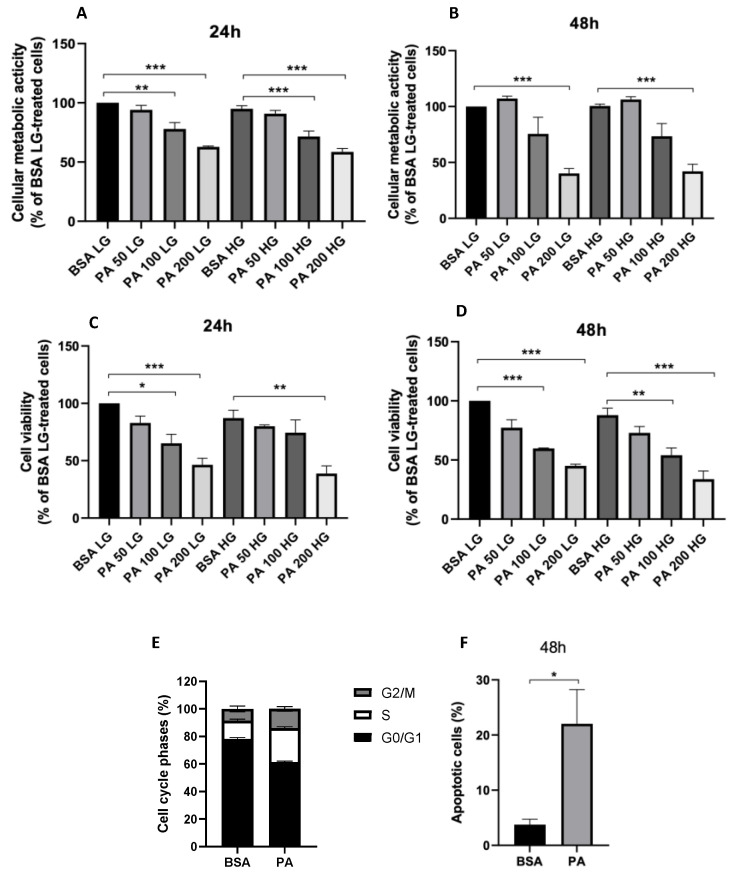
Palmitic acid (PA)-induced cytotoxicity. ARPE-19 cell metabolic activity (**A**,**B**) and viability (**C**,**D**) assessed after 24 (**A**,**C**) and 48 h (**B**,**D**) incubation with low glucose (LG, 5 mM glucose) or high glucose concentration (HG, 13 mM glucose) in the presence or absence of 50, 100 and 200 µM PA. Cell cycle (**E**) and apoptosis (**F**) of ARPE-19 cells in response to 48 h treatment with bovine serum albumin (BSA) or 200 µM PA. Results are reported as mean ± SEM of at least three independent experiments. * *p* < 0.05, ** *p* < 0.01, *** *p* < 0.001.

Hyperglycemia and increased circulating saturated free fatty acids are two key features of type 2 diabetes and are both fundamental in underpinning diabetes complications, including diabetic retinopathy [33]. Particularly, PA not only represents one of the most abundant circulating fatty acids in patients affected by type 2 diabetes [34] but has also been widely shown to exert lipotoxic effects in a variety of cell types [5,6,7]. In light of this, PA may play a pivotal role in disrupting the retinal pigmented epithelium, thereby contributing to diabetic retinopathy. For this reason, in the first set of experiments, it was evaluated if PA, alone or in combination with high glucose level, was able to exert a cytotoxic effect on ARPE-19. Our results showed that, while high glucose concentration alone did not affect cellular metabolic activity (Figure 2A,B) and cell viability (Figure 2C,D), PA induced a drop in both these parameters (Figure 3A–D) in a dose-dependent manner at both time points investigated (24 and 48 h after treatment), with a significant effect already being detected using 100µM PA. Remarkably, high glucose levels did not potentiate the lipotoxic effects of PA, both in terms of cellular metabolic activity (Figure 3A, B) as well as cell viability (Figure 3C,D).

Considering the decrease in cell number observed in Arpe-19 cells after the treatment with PA, we next tried to verify if this effect was due to a cytostatic or to a cytotoxic action. The cell cycle analysis revealed that PA, used at a concentration 200 µM, did not induce a blockade on it, but, at most, a slight increase in S phase after 48 h of treatment (*p* < 0.001, Figure 3E). Conversely, PA exerted a significant apoptotic effect at the same time point (Figure 3F).

### 3.2. Effect of PA on Oxidative Stress and Mitochondrial Morphology

**Figure 4:**
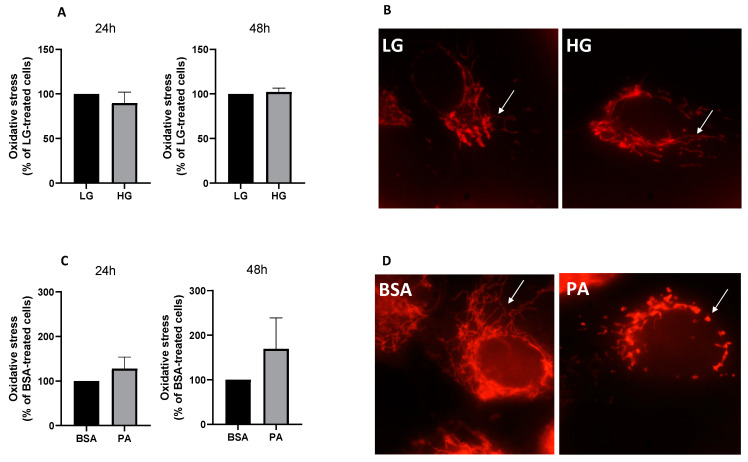
The effect of palmitic acid (PA) on oxidative stress and mitochondrial dynamics. ARPE-19 reactive oxygen species generation in response to 24 or 48 h incubation with low glucose (LG, 5 mM glucose) or high glucose concentration (HG, 13 mM glucose) (**A**) and bovine serum albumin (BSA) or 200 µM PA (**C**). Representative images of ARPE-19 mitochondria stained with MitoTracker^TM^ Red CMXRos following 48 h treatment with LG or HG (**B**) and BSA or PA (**D**). Results are reported as mean ± SEM of at least three independent experiments.

To elucidate the cellular mechanisms underpinning the cytotoxic effects of PA, mitochondrial dynamics and oxidative stress were investigated, confirming, first of all, that the ability of nutrient overload to elicit a cytotoxic insult, at least in the short term, was PA-dependent, with high glucose concentration failing to induce oxidative stress, both at 24 and 48 h (Figure 4A). In line with the close association between oxidative stress and mitochondrial fragmentation [35], high glucose concentration also did not induce mitochondrial fission (Figure 4B). On the contrary, PA tended to increase cellular ROS levels after 48 h of treatment (Figure 4C) and promoted mitochondrial fragmentation (Figure 4D).

### 3.3. Characterization of TRAIL Receptors and Effect of Recombinant TRAIL on PA-Induced Cytotoxicity

**Figure 5:**
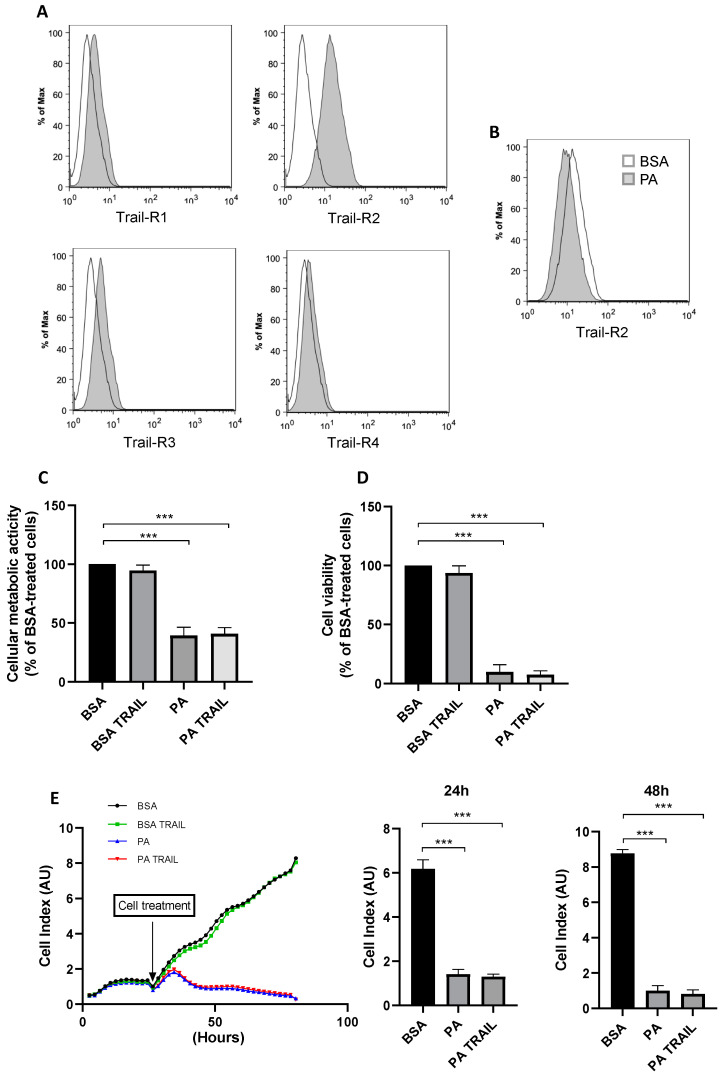
Surface expression of tumor necrosis factor (TNF)-related apoptosis-inducing ligand (TRAIL) receptors (Trail-R) and effect of TRAIL on palmitic acid (PA)-induced cytotoxicity. Representative expression of Trail-R at baseline (**A**) and after 48 h of treatment with Bovine serum albumin (BSA) or PA in ARPE-19 cells (**B**); cellular metabolic activity (**C**) and cell viability (**D**) after 48 h of treatment with BSA or PA in the presence or absence of TRAIL; cell density represented as time course or bar graphs (**E**) reporting ARPE-19 cell index after 24 and 48 h exposure to BSA or PA in the presence or absence TRAIL. Results are reported as mean ± SEM of at least three independent experiments. *** *p* < 0.001.

TRAIL can act as an inducer of apoptosis on tumor cells or as a positive modulator of cell-cycle progression in a cell-type-dependent manner [36]. Furthermore, downregulation of soluble TRAIL may be related to inflammation and angiogenesis in proliferative diabetic retinopathy [28]. Thus, in order to evaluate whether recombinant TRAIL may target ARPE-19 to modulate PA-induced cytotoxicity, the surface expression of the four TRAIL transmembrane receptors (TRAIL-R1, -R2, -R3, -R4) in this cell line was first investigated. As shown in Figure 5A, TRAIL-R2, as opposed to TRAIL-R1, TRAIL-R3 and TRAIL-R4, which were barely detectable or totally absent, was the only one expressed on the cell surface of ARPE-19 cells.

It was next assessed whether TRAIL-R2 expression could be modulated in response to the lipotoxic effect exerted by PA, verifying that the PA challenge did not induce an upregulation of TRAIL-R2 expression on ARPE-19 cells but only a slight decrease after 48 h treatment (Figure 5B). Additionally, despite ARPE-19 expressing TRAIL-R2, TRAIL treatment, used in a range 100–1000 ng/mL, did not affect ARPE-19 cell viability or metabolic activity (Figure S1). Moreover, the contemporary treatment with TRAIL and PA for 48 h, following the experimental design of Figure 1A, did not potentiate PA-induced cytotoxicity in terms of cell viability and cellular metabolic activity, nor did it protect cells from its effects (Figure 5C,D). As shown in Figure 5E, PA, but not TRAIL, promoted a reduction in the cell index and, therefore, cell density, both at 24 and 48 h of treatment. In agreement with the inability to counteract PA-induced cytotoxicity, TRAIL contemporary treatment also did not counteract the increase in cellular ROS levels, nor did it prevent the mitochondrial fragmentation promoted by PA (data not shown).

### 3.4. Characterization of the Cytotoxic Insult of PA after Its Removal

**Figure 6:**
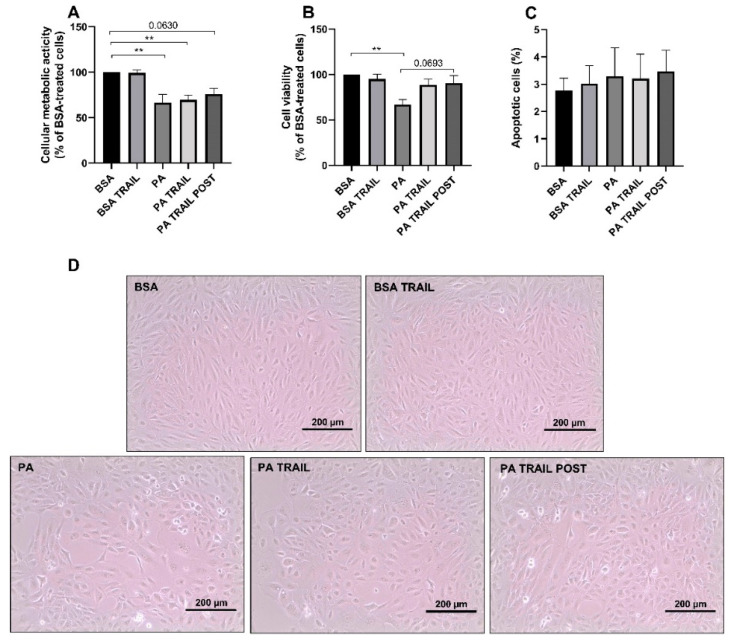
Effect of tumor necrosis factor (TNF)-related apoptosis-inducing ligand (TRAIL) on palmitic acid (PA)-induced cytotoxicity in the recovery experiments. ARPE-19 cell metabolic activity (**A**), viability (**B**) and apoptosis (**C**) assessed after 72 h incubation in growth media carried out after the removal of the following treatments: bovine serum albumin (BSA) in the presence or absence of TRAIL or PA in the presence or absence of TRAIL. A subset of PA-treated cells was exposed to TRAIL for 72 h following treatment removal (PA + TRAIL POST). In (**D**), representative bright-field images of ARPE-19 exposed to the above treatments. Results are reported as mean ± SEM of at least four independent experiments. ** *p* < 0.01.

To evaluate whether ARPE-19 cells were able to recover from PA-induced cytotoxicity and if this could be facilitated by TRAIL, PA was removed from the cell culture media and cells were allowed to grow for an additional 72 h, as outlined in Figure 1B,C. Cellular metabolic activity and cell viability remained significantly lower for cells treated with PA compared to those exposed to BSA (Figure 6A,B). However, the deleterious effect of PA on the number of viable cells was attenuated by TRAIL, both when used in combination with PA and then removed as well as when added following the 48 h PA challenge (Figure 6B). Indeed, in the latter groups, the amount of viable cells was not significantly lower with respect to control cells (Figure 6B). Furthermore, when TRAIL was added to the cells previously exposed to PA, it tended to increase the number of viable cells compared to cells in which PA was substituted by growth media. Moreover, the percentage of apoptotic cells was not affected by any of the treatments, indicating that the removal of PA was sufficient to counteract the increase in apoptotic cells observed after 48 h of treatment (Figure 6C). These data were confirmed by the morphological analysis of cells treated, as described above (Figure 6D).

### 3.5. Characterization of the Molecular Mechanisms Underlying the Persistence of PA-Induced Cytotoxicity and the Protective Effect of TRAIL

**Figure 7:**
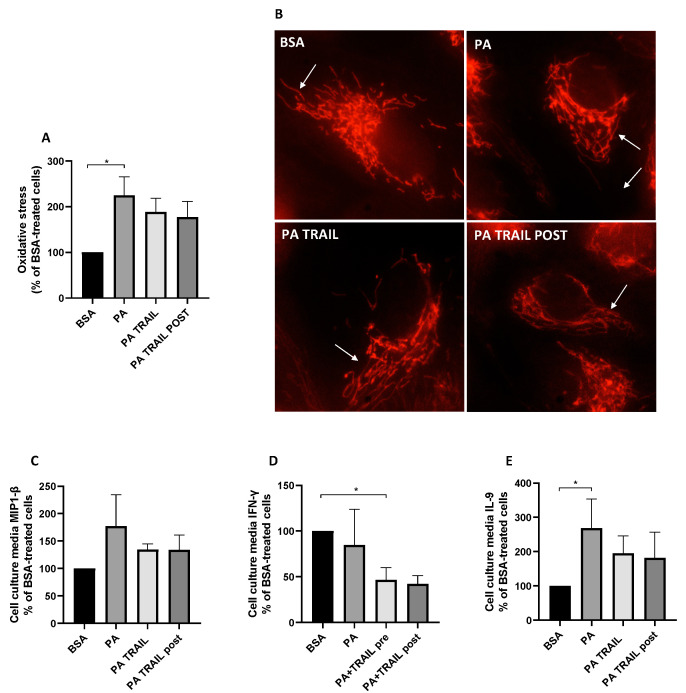
Effect of tumor necrosis factor (TNF)-related apoptosis-inducing ligand (TRAIL) on oxidative stress, mitochondrial dynamics and inflammation in the recovery experiments. ARPE-19 reactive oxygen species generation (**A**), representative images of ARPE-19 mitochondria stained with MitoTracker^TM^ Red CMXRos (**B**) and MIP 1-β (**C**), IFN-γ (**D**), as well as IL-9 (**E**) secretion assessed after 72 h incubation in growth media carried out after the removal of the following treatments: bovine serum albumin (BSA) in the presence or absence TRAIL, palmitic acid (PA) in the presence or absence of TRAIL. A subset of PA-treated cells was exposed to TRAIL for 72 h following treatment removal (PA + TRAIL POST). Results are reported as mean ± SEM of at least four independent experiments. Results are reported as mean ± SEM of at least three independent experiments. * *p* < 0.05.

To shed light on the retention of PA cytotoxic insult and the ability of TRAIL to counteract these effects, we investigated whether these effects were linked with the modulation of oxidative stress, mitochondrial morphology and inflammation. The cells treated with PA, despite its removal, displayed a persistent increase in cellular ROS with respect to BSA-treated cells, an effect that was slightly lowered by the co-treatment with TRAIL or the administration of TRAIL following the PA challenge (Figure 7A). On the contrary, mitochondrial morphology was completely restored following the PA removal, with ARPE-19 previously treated with PA displaying branched and fused mitochondria independently of the presence of TRAIL (Figure 7B). In agreement with the close association between oxidative stress and inflammation [37], we next analyzed the secretion of 27 cytokines/chemokines related to inflammation. Among these, cells treated with PA displayed an increase in MIP 1-β secretion, albeit not statistically significantly (*p* = 0.0715), whereas this effect was attenuated in cells treated with TRAIL, independently of whether this was used simultaneously with PA or added afterwards (Figure 7C). Similarly, the cells treated with TRAIL in combination with PA or with TRAIL following the PA challenge secreted less IFN-γ compared to cells previously treated with PA only (Figure 7D). Finally, the withdrawal of PA elicited a significant increase in IL-9 secretion in comparison to control cells and, on the contrary, this effect was absent in ARPE-19 cells in which TRAIL was used concomitantly with or added following PA removal (Figure 7E). All the other analyzed cytokines, when detectable, did not show a significant/tending-to-significant modulation.

## 4. Discussion

The data presented herein provide evidence on the lipotoxic effect perpetrated by PA in an in vitro model of human retinal pigment epithelial cells. Particularly, the results of this study indicate that the removal of this long-chain saturated fatty acid is instrumental for the cell to recover from its lipotoxic insult. This paradigm also holds true for TRAIL administration to ARPE-19 cells. Indeed, the ability of TRAIL to increase the number of viable cells and, therefore, exert a trophic effect, only appeared evident following the removal of PA.

PA is well known for its lipotoxic effects in a variety of cell models, ranging from neurons to myotubes [6,30,38,39]. Despite some studies having investigated the effect of PA on ARPE-19 [12,13], no report to date has fully characterized its lipotoxic effect on this cell model nor the capacity of these cells to recover from this metabolic insult. Hyperglycaemia and increased circulating saturated free fatty acids are two key features of T2DM and are both central in the pathogenesis of diabetes complications, including diabetic retinopathy [33]. In light of this, an important aspect that was investigated as part of this study, in the context of diabetic retinopathy, was the cytotoxic effect of high glucose concentration in combination with the saturated fatty acid PA on ARPE-19 cells. In this regard, at least in the short term, up to 48 h, high glucose concentration did not result in ARPE-19 cytotoxicity nor potentiate the effect of PA. This indicates that, in our experimental model, at least in the short term, PA represents a more detrimental metabolic insult compared to high glucose concentration. Despite this, high glucose concentration was previously shown to induce cytotoxicity [12]. However, this effect was elicited by 30 mM glucose, which represents a supraphysiological concentration, even for the diabetic condition, whereas in the present study, the concentration of glucose was maintained at 13 mM to more closely mimic diabetic hyperglycemia [40]. However, it is undeniable that even mild hyperglycemia, if sustained in the long term, comes with severe consequences, including those induced by protein glycation and the formation of advanced glycation end products, which substantially contribute to diabetic retinopathy and other diabetes complications [41].

In an attempt to analyze the cellular mechanisms at the basis of PA-induced lipotoxicity, we verified that the drop in cell viability and cellular metabolic activity elicited by PA itself did not rely on the inhibition of the cell cycle. Instead, PA did not promote a decrease in the number of cells in phase G0/G1 while increasing those in the S phase. Although this effect was surprising, especially because it occurred in parallel with a decrease in cellular metabolic activity and viability, it might be explained by the ability of the cells to sense an increase in energy availability in the form of PA. This excess of energy, in turn, may have pushed the cells towards the S phase of the cell cycle. Thus, rather than a cytostatic effect, the drop in cell viability and cellular metabolic activity elicited by PA in ARPE-19 cells appeared to be driven by apoptosis. Furthermore, lipotoxicity was not only witnessed by a decrease in cell viability and density, but also by an increase in mitochondrial fragmentation. Indeed, mitochondrial fragmentation represents a biomarker of environmental stress [42], with PA acting as the exogenous stressor in the case of the present study. Additionally, mitochondrial dynamics and function are tightly linked, with an increase in mitochondrial fission (i.e., fragmentation), leading to a drop in mitochondrial function [43]. In light of this, considering the pivotal role of mitochondrial function in fatty acid catabolism, the increase in PA availability in the face of impaired mitochondrial fatty acid oxidation may exacerbate the lipotoxic effect of lipid overload. In these conditions, PA may be funneled towards the synthesis of lipotoxic lipid species, which may not only be the consequence of mitochondrial fragmentation and dysfunction but also the cause [44]. The production of ROS is another key mechanism underlying the lipotoxic effect of PA in ARPE-19 apart from representing a key pathogenetic feature of diabetic retinopathy [45]. Furthermore, the production of ROS is closely linked to mitochondrial fragmentation. Indeed, while mitochondrial fragmentation promotes ROS production, increased intracellular ROS trigger mitochondrial fragmentation [46]. Most importantly, inhibition of mitochondrial fission was reported to attenuate the cellular damage induced by oxidative stress in ARPE-19 cells [47], further supporting the involvement of mitochondrial fragmentation as a potential driver of PA-induced cytotoxicity. In line with this, in the present study, high glucose concentration not only did not promote mitochondrial fragmentation but also failed to induce an increase in ROS production, which is in line with the lack of cytotoxicity observed in response to 13 mM glucose.

Soluble TRAIL levels in the conjunctival sac fluid [26] and in vitreous samples [28] have been reported to be significantly decreased in patients with diabetic retinopathy and to protect against diabetes [19]. Additionally, ARPE-19 cells express the TRAIL-R2, which strengthens the rationale of using TRAIL as a potential therapeutical tool to counter the lipotoxic effect of TRAIL, since TRAIL was previously reported to exert both pro-apoptotic and pro-survival effects, depending on the cell type and the context [48]. However, when used in the presence of PA, TRAIL failed to inhibit its cytotoxic effect in terms of cellular metabolic activity, cell viability and density, which is in agreement with the inability of TRAIL to counter mitochondrial fragmentation and ROS production. A potential explanation for the incapacity of TRAIL to rescue PA-induced cytotoxicity is due to the fact that the magnitude of the cytotoxic effect exerted by PA overcomes the cytoprotective effects of TRAIL, including its trophic effects [49]. To test this possibility, the pro-survival effects of TRAIL were tested following the removal of the cytotoxic insult. TRAIL was able to increase the number of viable cells only when PA was removed from the cell culture media, with this effect occurring both when TRAIL was used alongside PA as well as added following the removal of the treatments. This suggests that the removal of the cytotoxic insult elicited by PA is pivotal for TRAIL to exert its trophic effect.

In terms of the mechanisms underpinning the trophic effect of TRAIL following the removal of PA from the cell culture media, these appear to rely upon inflammation and ROS production, but not on mitochondria fragmentation. Indeed, the effect of PA on mitochondrial fragmentation is transitory and fully reversible, suggesting that mitochondria recover their morphology independently of the cell treatment with TRAIL. Instead, oxidative stress is long lived and persists, despite PA removal, suggesting that the ARPE-19 cell line retained at least some features of the metabolic insult mediated by this long-chain saturated fatty acid. Interestingly, TRAIL used both alongside PA and after its withdrawal tended to decrease ROS production, an effect that may explain the ability of TRAIL to facilitate cell proliferation in the recovery phase (i.e., following the removal of the insult). These effects are in agreement with the previously reported capacity of TRAIL to inhibit oxidative stress induced by pro-inflammatory stimuli in human aortic endothelial cells [50]. PA has also been widely reported to trigger inflammatory responses, which are paralleled by the induction of oxidative stress [51,52], with both pathophysiological mechanisms interacting with one another [53], potentially contributing to sustaining lipid-overload-induced metabolic insult. Interestingly, the cytokine secretory profile of ARPE-19 cells follows the same pattern as oxidative stress, with cells treated with PA displaying a pro-inflammatory secretory profile, despite its removal. Remarkably, TRAIL, in agreement with other reports [16,54,55], exerted a putative anti-inflammatory effect and mitigated the inflammatory response promoted by PA. This further suggests that the mitigation of the inflammation–oxidative stress axis may be at the basis of the ability of TRAIL to rescue cell proliferative capacity following exposure to PA. Not surprisingly, the activation of the oxidative stress–inflammation axis is a pivotal pathogenetic mechanism underpinning the cytotoxic effects elicited by metabolic fuel overload in the context of diabetic retinopathy [56,57,58]. Thus, TRAIL appeared to facilitate the recovery of ARPE-19 following the removal of PA, possibly via the inhibition of oxidative stress and of specific inflammatory pathways. On the contrary, cells treated with PA alone retained the pathophysiological phenotype triggered by this long-chain saturated fatty acid, which was marked by oxidative stress and inflammation.

## 5. Conclusions

It must be acknowledged that the in vitro data obtained on an immortalized cell line cannot be easily translated into clinics and that the findings of this study, relative to the therapeutic potential of TRAIL, need to be replicated in in vivo models. Nevertheless, these data suggest that TRAIL is effective in restoring RPE cell survival, but this effect, to be manifested, requires that the lipotoxic stimulus is reduced/abrogated. In a therapeutic perspective, this means that both normalization of the lipid metabolic profile and practices aimed to elevate the levels of TRAIL, both in the general circulation and locally in the retina, might be beneficial in the early onset of DR when the proliferative stage of diabetic retinopathy has not taken place yet.

## Data Availability

Data is contained within the article and supplementary material.

## Supplementary Materials

The following are available online at https://www.mdpi.com/article/10.3390/antiox11122340/s1, Figure S1: Cellular metabolic activity and viability in response to increasing TRAIL concentrations.
