# Supplementary material for: Palmitic Acid Induced a Long-Lasting Lipotoxic Insult in Human Retinal Pigment Epithelial Cells, which Is Partially Counteracted by TRAIL
Source: Antioxidants (Basel). 2022 Nov 26;11(12):2340. doi: 10.3390/antiox11122340 (PMC9774631; doi:10.3390/antiox11122340)
Supplement: Supplementary file 1 [file antioxidants-11-02340-s001.zip › antioxidants-2000406-supplementary.pdf]

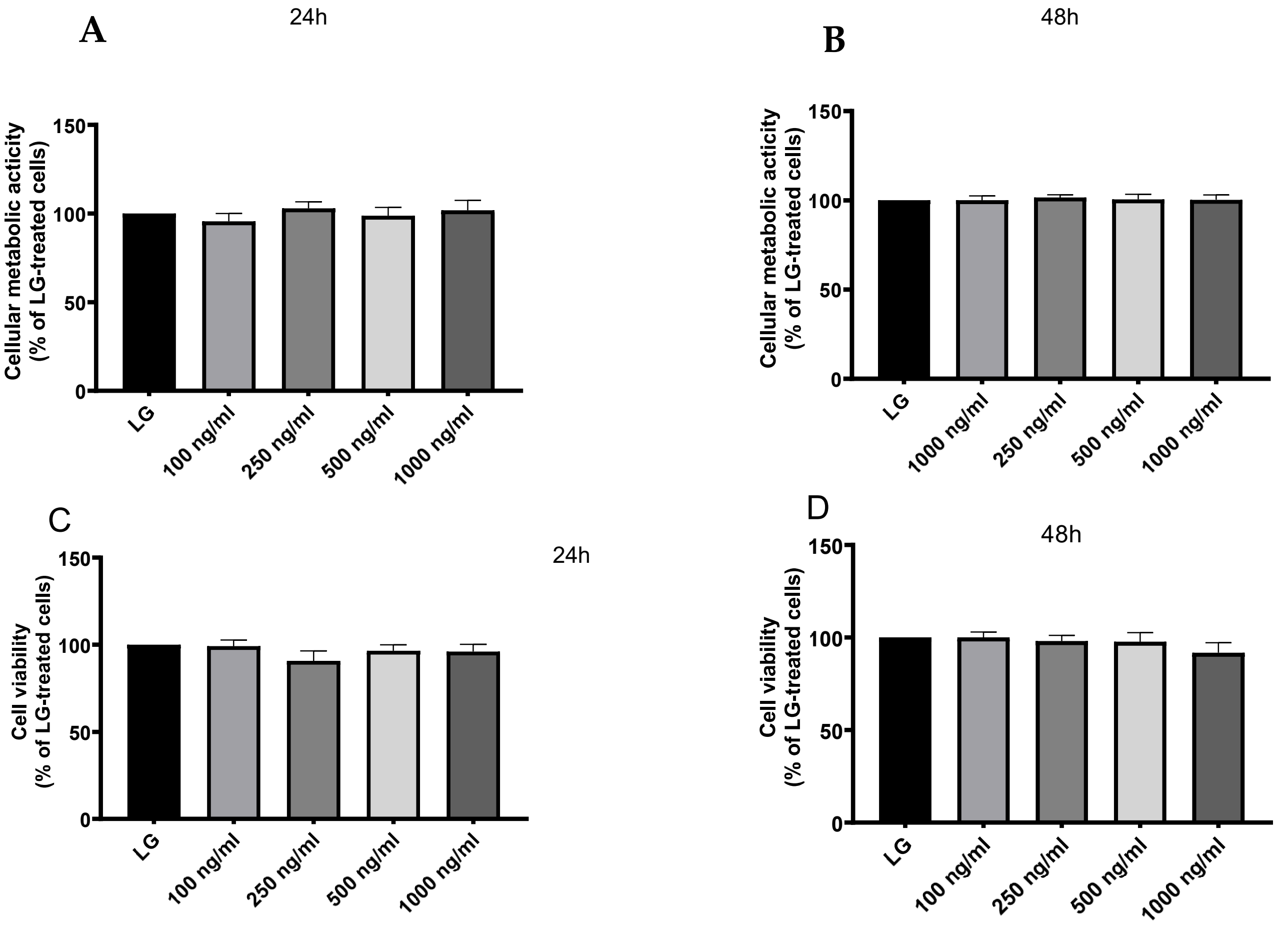

Supplementary Figure S1. Cellular metabolic activity and viability in response to increasing TRAIL concentrations: 100, 250, 500 and 1000 ng/ml. ARPE-19 cell metabolic activity (A, B) and cell viability (C, D) assessed after 24 (A, C) and 48 hours (B, D) incubation with increasing TRAIL concentrations. Data are expressed as mean ± SEM of two independent experiments, each performed in triplicate.
